# Anatomic basis for a new ultrasound‐guided, mini‐invasive technique for release of the deep transverse metatarsal ligament

**DOI:** 10.1002/ca.23692

**Published:** 2020-10-12

**Authors:** Gabriel Camunas Nieves, Alejandro Fernández‐Gibello, Simone Moroni, Ruben Montes, Javier Márquez, Mario Suárez Ortiz, Teresa Vázquez, Fabrice Duparc, Bernhard Moriggl, Marko Konschake

**Affiliations:** ^1^ Clínica Vitruvio Madrid Spain; ^2^ Faculty of Health Sciences, Department of Podiatry University of La Salle Madrid Spain; ^3^ Faculty of Health Sciences at Manresa, Department of Podiatry Universidad de Vic ‐ Universidad Central de Catalunya (UVic‐Ucc) Barcelona Spain; ^4^ Faculty of Health Sciences, Department of Podiatry Universidad Católica de Valencia Valencia Spain; ^5^ Clínica Podosalud Madrid Spain; ^6^ Anatomy and Embryology Department School of Medicine, Complutense University of Madrid Madrid Spain; ^7^ Laboratory of Anatomy, Faculty of Medicine Rouen‐Normandy University Rouen France; ^8^ Department of Anatomy, Histology and Embryology Institute of Clinical and Functional Anatomy, Medical University of Innsbruck (MUI) Innsbruck Austria

**Keywords:** deep transverse metatarsal ligament, minimally invasive, Morton's neuroma, ultrasound

## Abstract

**Introduction:**

Morton's neuroma is an entrapment neuropathy of the third common plantar digital nerve, caused by the deep transverse metatarsal ligament (DTML). Minimally invasive or percutaneous surgery is a very common procedure, but surgical effectivity of this technique remains controversial. The goal of our study was to prove the effectiveness and safety of a new ultrasound‐guided technique for DTML‐release in a cadaver model.

**Materials, Methods, and Results:**

The DTML was visualized in 10 fresh frozen donated body to science‐feet (eight male and two females, five left and five right) using an US device (GE Logic R7; 13 MHz linear probe, Madrid, Spain). Consecutively, minimally invasive ultrasound‐guided surgery was performed. Exclusion criteria of the donated bodies to science were previous history of forefoot surgery and space occupying mass lesions. The complete release of the ligament was achieved in all specimens without damage of any important anatomical structures as proven by anatomical dissection.

**Conclusions:**

The results of this study indicate that our novel approach of an ultrasound‐guided release of the DTML is safer and more effective compared to blind techniques. The DTML could reliably be visualized and securely cut through a dorsal, minimally invasive surgical incision of only 2 mm.

## 1 INTRODUCTION

Morton's neuroma (MN) was initially mentioned by Civinini in 1835 (Civinini, 1835). Later—in 1845—Durlacher described the clinical symptoms and finally in 1876, Thomas George Morton reported the condition of metatarsalgia as a “peculiar and painful affliction of the fourth metatarsophalangeal articulation” (Adams, 2010; Civinini, 1835; Di Caprio, Meringolo, Shehab Eddine, & Ponziani, 2018; Durlacher, 1845; Matthews, Hurn, Harding, Henry, & Ware, 2019; Morton, 1876).

MN is not a true neoplastic or proliferative process due to its degenerative pathogenesis with histologically verified demyelination of the nerve fibers, fibrosis of the epi‐ and endoneurium including the so‐called Renaut bodies and densely packed whorls of collagen (Adams, 2010). Many different theories have been postulated to explain the pathogenesis of MN: repeated microtrauma, chronic traction damages, ischemia of the vasa nervorum, a secondary neurofibrosis after inflammatory intermetatarsal bursitis, increased tension in the foot fasciae and, currently widely accepted, an entrapment neuropathy of the third common plantar digital nerve due to an external compression by the deep transverse metatarsal ligament (DTML) (Stecco et al., 2015; Valisena, Petri, & Ferrero, 2018).

The incidence of chronic entrapment of a common plantar digital nerve is highest in the third intermetatarsal space (i.e., MN), followed by the second space (i.e., Hauser's neuroma) and least at both, the fourth (i.e., Iselin's neuroma) and the first space (i.e, Heuter's neuroma) (Larson, Barrett, Battiston, Maloney, & Dellon, 2005; Matthews et al., 2019). The female‐to‐male ratio reported in the literature is 4:1 (Di Caprio et al., 2018). Clinically, patients complain of burning metatarsal pain, often radiating to the toes, and sharp shooting sensations of pain (Di Caprio et al., 2018). Pain is exacerbating when the patient wears tight shoes or high heels, which leads to a narrow intermetatarsal space, augmented plantar metatarsal ground reaction forces and lastly to an extension of the metatarsophalangeal joint—resulting in compression of the common digital plantar nerve beneath the DTML (Di Caprio et al., 2018). Apart from clinical diagnosis considered as the gold standard, imaging techniques such as ultrasonography (US) or MRI, were cited for detection of a common plantar digital nerve entrapment. Notably, both MRI and US were rated just equally or even less accurate compared to physical examination (Claassen et al., 2014; Di Caprio et al., 2018; Lee et al., 2007; Sharp, Wade, Hennessy, & Saxby, 2003). Nevertheless, especially in skilled hands, US has its own and well documented advantages (Pastides, El‐Sallakh, & Charalambides, 2012).

We are, to the best of our knowledge, not aware of studies dealing with US‐visualization of the DTML as a basis for minimally invasive decompression surgery in the given context. Therefore, the goal of our study was to develop a new, landmark‐based, ultrasound‐guided, minimally invasive technique for a DTML‐release for Morton's neuroma.

### 1.1 Normal anatomy of the DTML and its topographical relationships

**FIGURE 1:**
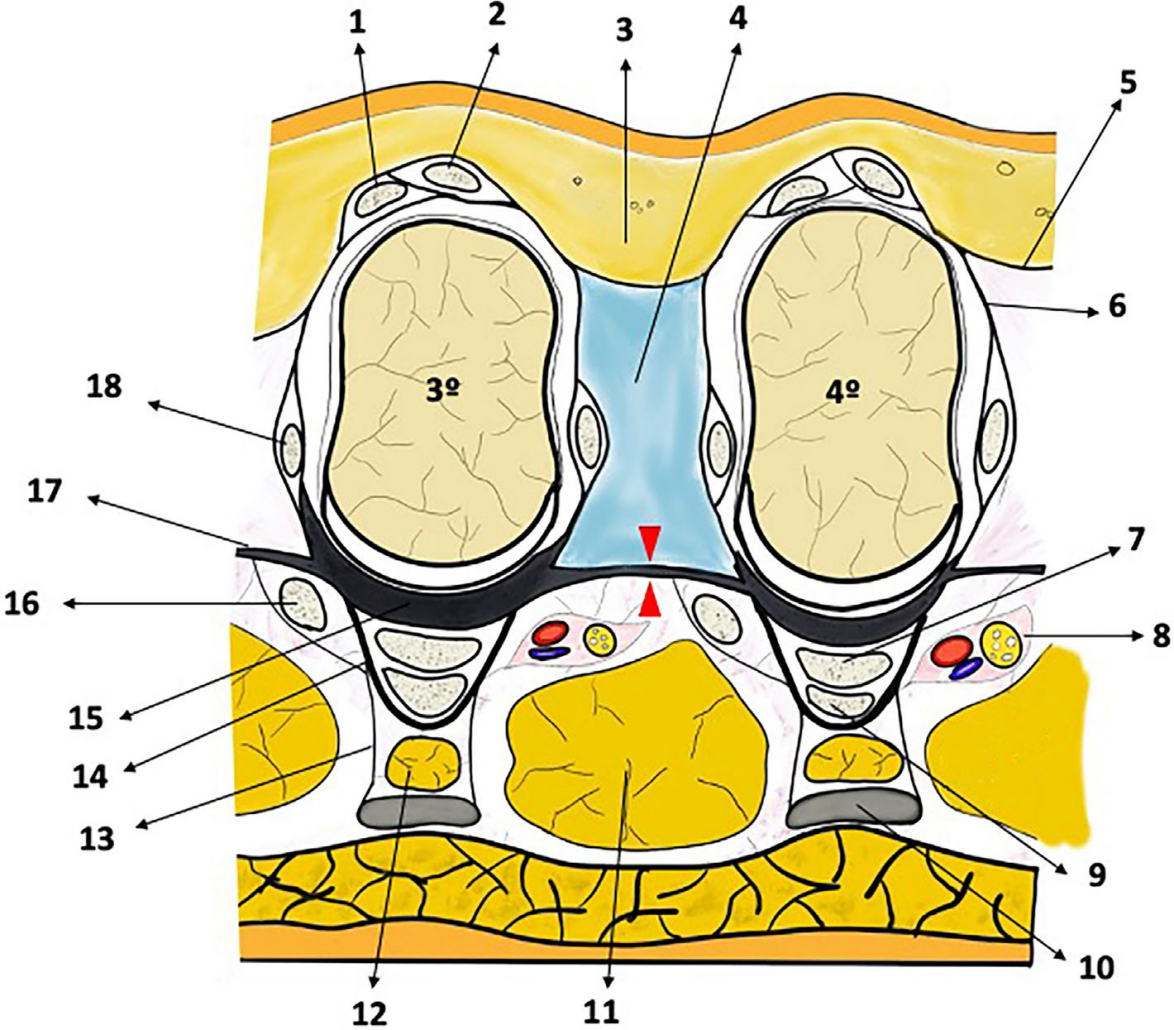
Frontal plane view of the intermetatarsal space at the level of third and fourth metatarsal heads (3°; 4°) showing topographical relationships. Red arrowheads: transection site of DTML (17), 1: extensor digitorum brevis tendon, 2: extensor digitorum longus tendon, 3: triangular adipose‐fascial complex carrying superficial nerves and vessels, 4: intermetatarso‐phalangeal bursa, 5: deep dorsal fascia, 6: vertical lamina of extensor aponeurosis, 7: flexor digitorum brevis tendon, 8: common digital plantar neurovascular bundle, 9: flexor digitorum longus tendon, 10: longitudinal fascicle of plantar aponeurosis, 11: plantar monticuli, 12: pretendinous fat compartment, 13: vertical extension of plantar aponeurosis forming the pretendon flexor space for adipose cushion, 14: Fibrous flexor tendon sheath**s**, 15: plantar plate, 16: tendon of lumbrical muscle, 17: DTML, 18: tendon of interosseous muscle. Figure based on the work of Stecco et al. (Stecco et al., 2015) [Color figure can be viewed at wileyonlinelibrary.com]

The individually varying distance between the metatarsals is defined by two mostly transverse structures: the deep dorsal intermetatarsal fascia and the DTML (Figures 1 and 5). The latter in particular, helps to avoid too much splaying of the toes (Figures 1 and 5). The DTML is a mostly transverse band embedded in the plantar metatarsophalangeal joint capsule complex, which is composed of four parts connecting all heads of the metatarsal bones and heads of phalanges to form a functional unit.

Dorsally to the DTML a synovial bursa (the so‐called “intermetatarso phalangeal bursa”) can be found that protects both metatarsal heads and the tendons of the interosseous muscles from fraying. Sandwiched between the ligament and the plantar fat body (also called “plantar monticuli”) (Kelikian & Sarrafian, 2011), the common plantar neurovascular bundle can be found adjacent to the third plantar plate, while the tendon of the lumbrical muscle is situated more fibularly close to the fourth plantar plate (Stecco et al., 2015) (Figures 1 and 3a).

## 2 MATERIALS AND METHODS

All consecutive steps described below were performed in 10 fresh frozen feet of eight male and two female cadavers (five right and five left, aged between 65 and 80 years), which belong to the Body Donation Centre.

The individuals had given their written informed consent for their use for scientific purpose prior to death. According to National Law, scientific institutions (in general Institutes, Departments or Divisions of Medical Universities) are entitled to receive the body after death mainly by means of a specific legacy, which is a special form of last will and testament. No bequests are accepted without the donor having registered their legacy and been given appropriate information upon which to make a decision based upon written informed consent (policy of ethics); therefore, an ethics committee approval was not necessary (Konschake & Brenner, 2014; McHanwell et al., 2020).

The exclusion criteria for this study were previous history of forefoot surgery and space occupying mass lesions.

Equipment used: high‐frequency US‐system with a 13 MHz linear probe (General Electric Logic R7; 8 mHz, Madrid, Spain), a scalpel for minimally invasive surgery with a beaver 64 blade, and a buttoned probe.

### 2.1 “Step‐by step” approach

After US‐visualization of the DTML (Step I), we performed a landmark‐based, minimally invasive US‐guided surgical approach in all specimens (Step II), followed by anatomical dissections of the third intermetatarsal space (Step III) evaluating the effectiveness and safety of surgical release of the DTML.

#### 2.1.1 Step I: Anesthesia, US‐identification of the DTML and skin incision

To avoid alteration of the clarity of the US image due to preprocedural local anesthesia bolus in such small probe girth we recommend a proximal anesthesia procedure (e.g., US‐guided ankle blocks) or at tarsal‐metatarsal joint level.

**FIGURE 2:**
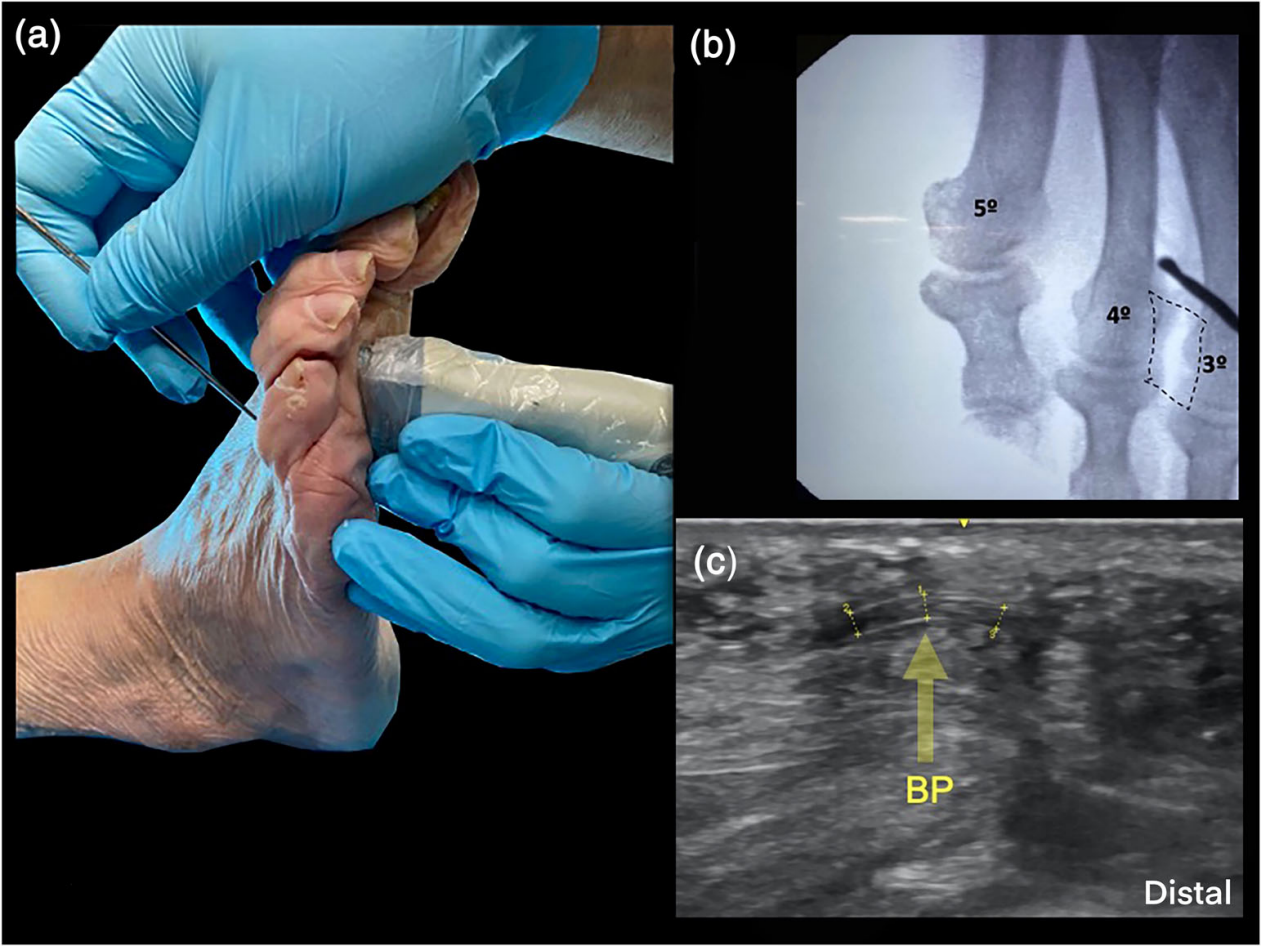
**(**a) The figure shows the positioning of the instruments—buttoned probe dorsal, US transducer plantar. (b) Fluoroscopy of the foot before the DTML release; dotted line shows approximate area of the DTML, buttoned probe also visible. (c) Longitudinal scan of the DTML: The buttoned probe (BP) pushes the ligament plantarly during the surgical procedure at the third intermetatarsal space [Color figure can be viewed at wileyonlinelibrary.com]

**FIGURE 3:**
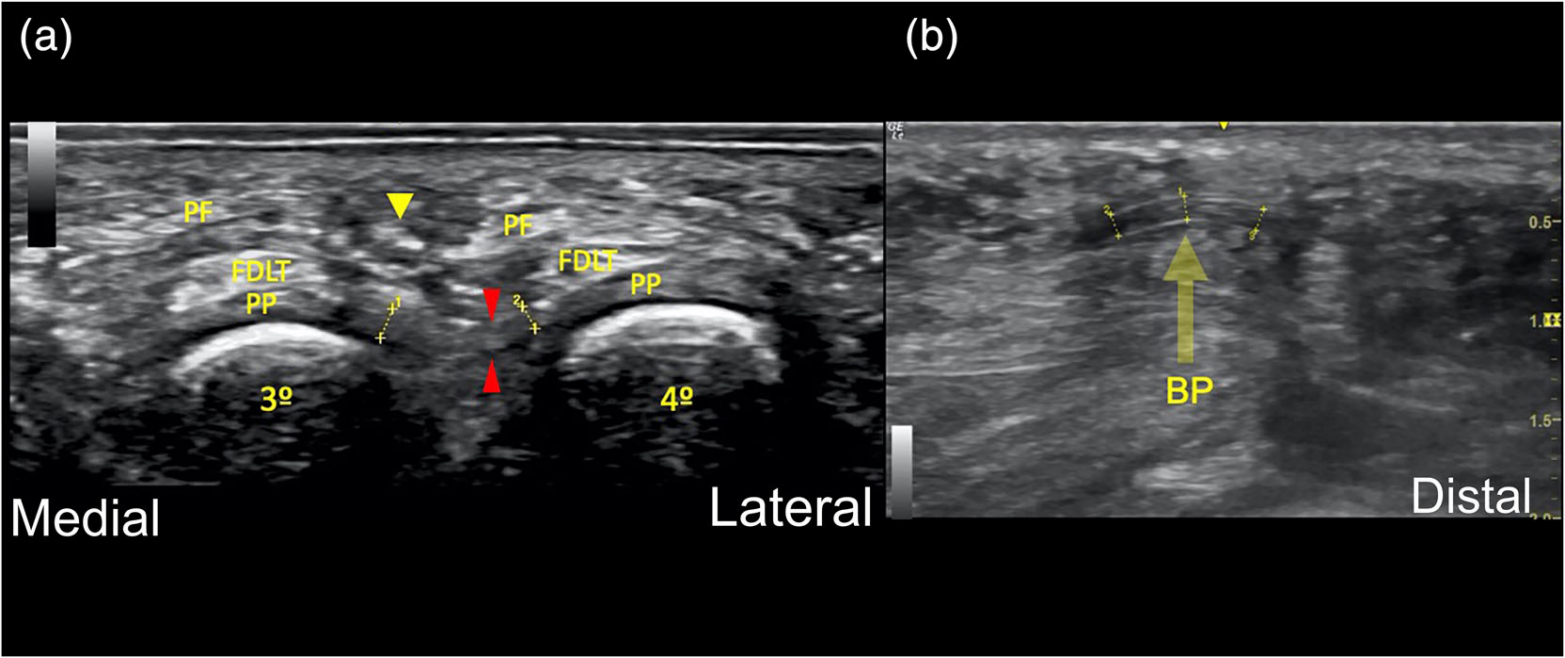
**(**a) From medial to lateral, (b) from proximal to distal: US‐appearance of the deep transverse metatarsal ligament (DTML) in two planes: the typical “white–dark–white” pattern of a ligament can be seen with hyperechoic borders (“white”), the substance of the rest of the ligament appears hypoechoic (“dark”) with just a few echogenic speckles. The mean thickness measured was 1.1 mm. (a) transverse scan of the DTML: red arrowheads (transection zone); plantar plate (PP); flexor digitorum longus tendon (FDLT); plantar aponeurosis (PF); yellow arrowhead (neurovascular bundle) (b) longitudinal scan of the DTML: The buttoned probe (BP) pushes the ligament plantarly during the surgical procedure [Color figure can be viewed at wileyonlinelibrary.com]

The US probe was positioned at the sole, between the heads of the respective metatarsals and in the long axis of the third intermetatarsal space for visualization of the DTML. As a control, the US probe was turned 90**°** to identify the ligament spanning between the plantar plates, and the position of the neurovascular bundle in its typical location (Figures 1 and 3a). To ease identification of the DTML in the longitudinal view through a marked movement, a buttoned probe was simultaneously placed at the dorsum of foot between the metatarsal heads and pushed plantarly (Nieto García, 2016) (Figures 1, 2, 3). The dorsal longitudinal skin incision was done with a scalpel close to the surgical neck of the fourth metatarsal head (the “surgical neck” being a constriction straight proximal to the head of the metatarsal bone, which is frequently seat of fractures).

#### 2.1.2 Step II: Surgical procedure under US‐guidance

After clear identification of DTML, we introduced the blade through the skin incision and advanced it gently toward the DTML under direct US visualization in plane. The ligament was then transected from proximal to distal. In order to verify the complete release of the DTML, the buttoned probe was introduced again, and its tip moved from proximal to distal. In case of incomplete transection (especially its most distal fibers), the step was repeated.

**FIGURE 4:**
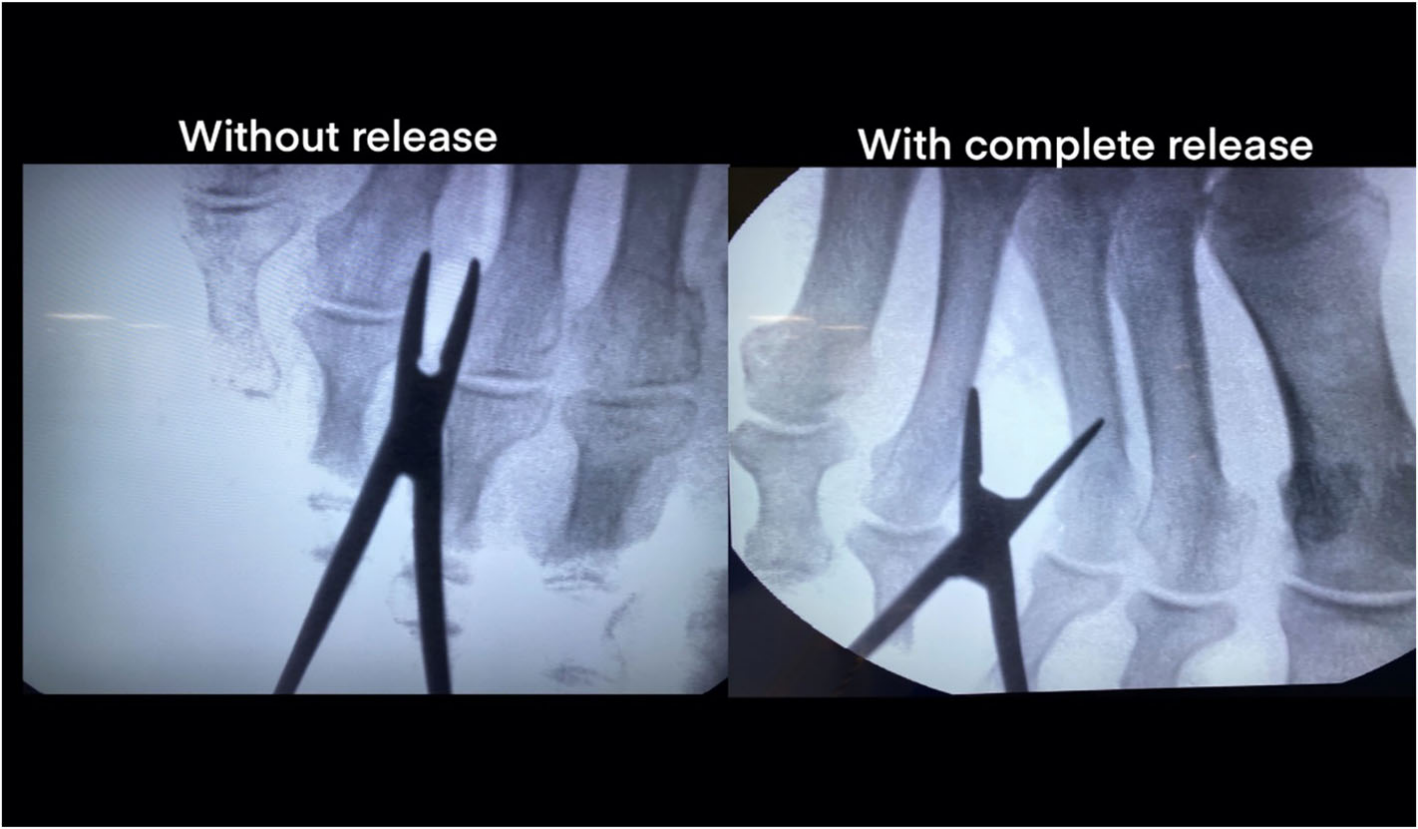
Confirmation of a complete release of the deep transverse metatarsal ligament (DTML) using fluoroscopy**—**left before, right after the ultrasound‐guided surgical release with widening the third intermetatarsal space [Color figure can be viewed at wileyonlinelibrary.com]

Prior to anatomical dissection, a retractor was inserted dorsally by enlargement of the initial incision in order to verify a widening of the intermetatarsal space under fluoroscopy (Figure 4).

#### 2.1.3 Step III: Anatomical dissection

**FIGURE 5:**
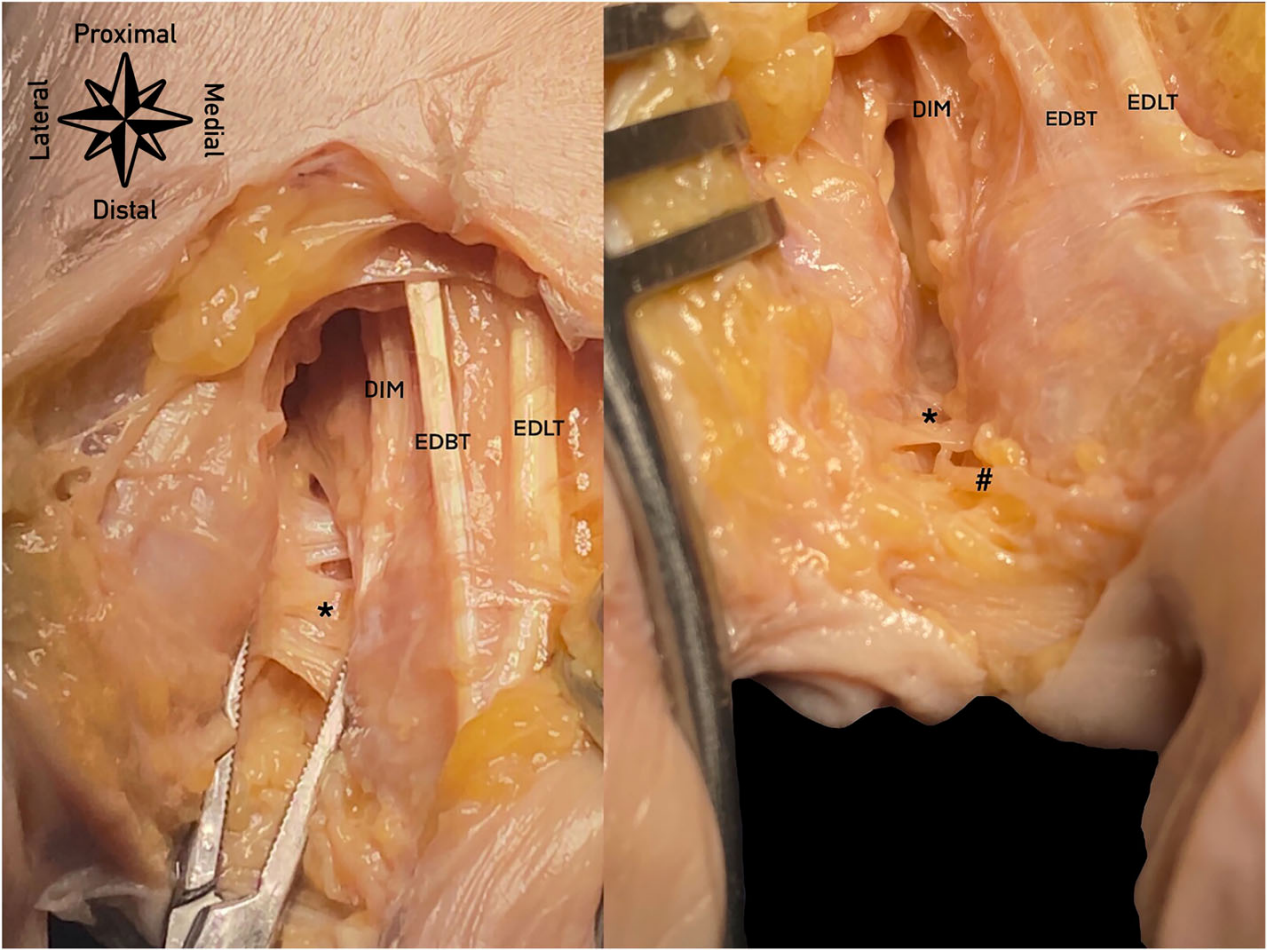
Anatomical specimen showing the DTML and its topographical situation. From lateral to medial: *DTML: deep transverse metatarsal ligament, #common plantar digital nerve, DIM: dorsal interosseous muscle, EDBT: extensor digitorum brevis tendon, EDLT: extensor digitorum longus tendon [Color figure can be viewed at wileyonlinelibrary.com]

An anatomical dissection of all 10 ft was finally made to verify a complete release of the DTML and to proof if no adjacent anatomical structures were injured, respectively: extensor expansion, interosseous muscles, lumbrical muscle, the collateral ligaments, plantar plate, vessels and nerves. For that, the widened incision for placing the first retractor as mentioned above, a further extension was done longitudinally, and another retractor inserted. Fat and connective tissue were removed carefully until we could visualize all important structures and the complete release of the DTML, respectively (Figures 1 and 5).

## 3 RESULTS

In all 10 ft, the DTML was successfully identified with US in two planes. **(**Figure 3a,b). With the transverse view, most important structures in the vicinity (i.e., the neurovascular bundle and plantar plates) were seen clearly (Figure 3a). The US‐appearance of the DTML was similar to the well‐known characteristics of a true ligament elsewhere in the locomotor apparatus: compared to sharply delineated hyperechoic borders (“white”) the substance of the rest of the ligament appears hypoechoic (“dark”) with just a few echogenic speckles. (Figure 3a,b) This “white–dark–white” pattern was constant in all of the 10 DTML monitored. The mean thickness measured was 1.1 mm. However, it has to be mentioned that, visualizing the nerve branches in this space is difficult; a lot of training and practical experience is mandatory.

The mean length of the incision necessary for our surgical approach was just 2 mm and thus the procedure may be defined as “ultra‐minimally invasive surgery” (Fernandez‐Gibello et al., 2019; Moroni, Zwierzina, et al., 2019; Moroni, Gibello, et al., 2019).

Complete release of the DTML was confirmed in all 10 ft without any injury of crucial adjacent structures.

## 4 DISCUSSION

In the current anatomical study, we describe a new, landmark‐based minimally invasive ultrasound‐guided surgical release of the DTML. The latter accounts for the majority of cases with compression of the third common digital nerve within its respective intermetatarsal space, leading to Morton's neuroma (Larson et al., 2005). Fabié et al. performed the same blind technique as de Prado (de Prado, Ripoll, & Golanó, n.d.; Fabié, Accadbled, Tricoire, & Puget, 2007). They could reach a complete and safe release in no more than 37.5% of their series (6 of 16 ft) (Fabié et al., 2007). They also described all anatomical structures being at risk during Morton's neuroma surgery (Fabié et al., 2007). Injury of the digital plantar arteries is also very common in 39% of open surgeries, an arteriotomy has occurred accidentally (Adams, 2010; Giakoumis, Ryan, & Jani, 2013). Therefore, it was about time for a new, US‐guided approach that might be safer than the reported “blind” techniques with their inherent risk of damage to anatomical structures (Fabié et al., 2007; Nieto García, 2016). Admittedly, a personal learning curve and profound anatomical knowledge is mandatory.

In podiatric surgery, minimally invasive blind approaches are common and, at least in short‐term, overall good outcomes regarding pain reduction were communicated (de Oliveira et al., 2019). However, in medium and long‐term, damages to tendons of lumbrical muscles, capsule ligaments and/or extensor hoods lead to deformities (Dalmau‐Pastor et al., 2014). Despite a correct release of the DTML without damaging nearby anatomical structures, Wang et al. (Wang et al., 2015) found a substantial instability in the lesser metatarsophalangeal joints in which the dorsiflexion stiffness was significantly reduced by a mean of 17% and the dorsal subluxation stiffness by 16%, respectively. For this reason, it is imperative to improve motor control of the “foot core system” by strengthening the plantar flexors to prevent future complications after decompression surgery (McKeon, Hertel, Bramble, & Davis, 2015). Injury of the digital plantar arteries is also very common in 39% of open surgeries, an arteriotomy has occurred accidentally (Adams, 2010; Giakoumis et al., 2013). According to Valisena et al. and Santiago et al. (Ruiz Santiago, Prados Olleta, Tomas Munoz, Guzman Alvarez, & Martinez Martinez, 2019; Valisena et al., 2018), results of surgical treatment for primary Morton's neuroma were superior to both, conservative regimen and therapy done by injections. Noteworthy, infiltrative treatments under US guidance seem to have a better outcome compared to blind injections (Ruiz Santiago et al., 2019; Valisena et al., 2018). In accordance with our study of an US‐guided surgical procedure, Ruiz Santiago et al. (2019) stated that using US‐guidance increases effectiveness and accuracy of interventional procedures. This is due to a real‐time visualization of important anatomical structures, decreasing the likelihood of injuries (Ruiz Santiago et al., 2019). As evaluated by US, it has been estimated that for interventional procedures such as corticosteroid injections, the cut‐off value of 6.3 mm is the threshold size of the MN (Park, Lee, Choi, & Kim, 2018).

Apart from interventional techniques, alternative nonsurgical options like manipulation and/or mobilization exist (Matthews et al., 2019). Their high evidence for a symptomatic pain reduction has been evaluated in a metanalysis by Matthews et al. (2019). According to the group of Stecco et al. (2015), this might be possible due to the chronic rigidity of the dorsal deep fascia of the foot, causing an increased stiffness of the intermetatarsal spaces. Furthermore, customized insoles with metatarsal and arch support can reduce pain during walking as described by de Oliveira et al. (2019).

On the other hand, both neurolysis and neurectomy have shown good and similar results in pain relief after Morton's neuroma procedures (Lu et al., 2020). Furthermore, O'Connor, Johnson, McCormick, and Klein (2016) demonstrated that clinical, intraoperative and histopathological diagnosis was in concordance in up to 100%. Therefore, histopathological examination of every excised neuroma after surgery might not necessary based on large series (O'Connor et al., 2016). Different researches concluded that minimally invasive surgery offers the same results as open surgeries. Newer publications in other fields of podiatric surgery (e.g., tarsal tunnel decompression) report even better outcomes and less adverse effects when performed under ultrasound guidance (Fernandez‐Gibello et al., 2019; Moroni, Gibello, et al., 2019). Therefore, a personal learning curve in the use of US and profound anatomical knowledge provided, we consider our reported ultrasound‐guided, minimally invasive as the first surgical option in selected patients. However, it has to be mentioned that visualizing all anatomical structures in this space might be difficult and training and practical experience is mandatory. More data of evidence‐based, clinical outcomes of our described procedure will be presented in consecutive studies in the near future.

## 5 CONCLUSION

Our new ultrasound‐guided, minimally invasive surgical approach for treatment of Morton's neuroma seems to be a safer and more effective technique in the release of the DTML as compared to previously reported blind techniques. The DTML shows the same and constant US‐features as any other true ligament in the human body. This enables its reliable visualization, which is fundamental to a safe, US‐guided transection that avoids injury to adjacent anatomical structures.

## CONFLICT OF INTEREST

The authors have nothing to declare, no conflict of interest.
